# Cutaneous and Lymphangitic Infection Caused by *Purpureocillium lilacinum* in Immunocompromised Patients: A Case Report with a Narrative Review of the Literature

**DOI:** 10.3390/life15091453

**Published:** 2025-09-17

**Authors:** Tommaso Lupia, Cristina Sarda, Francesca Canta, Marco Casarotto, Guido Accardo, Gabriele Roccuzzo, Nicole Macagno, Federica Gelato, Rebecca Senetta, Antonio Ottobrelli, Francesco Giuseppe De Rosa, Silvia Corcione, Simone Ribero, Pietro Quaglino, Paolo Fava

**Affiliations:** 1 Unit of Infectious Diseases, City of Health and Sciences, 10100 Turin, Italy; 2 Dermatology Clinic, Section of Dermatology, Department of Medical Sciences, University of Turin, 10100 Turin, Italy; 3 Department of Medical Sciences, Infectious Diseases, University of Turin, 10100 Turin, Italy; 4 Pathology Unit, Department of Oncology, University of Turin, 10100 Turin, Italy; 5 Hepatology Unit, AOU Città della Salute e della Scienza di Torino, University of Turin, 10100 Turin, Italy

**Keywords:** *Purpureocillium lilacinum*, *Paecilomyces lilacinus*, liver transplant, immunocompromised patients

## Abstract

Background: *Purpureocillium lilacinum* (*P. lilacinum*) is an emerging filamentous fungus known to cause opportunistic infections, particularly in immunocompromised patients. Formerly known as *Paecilomyces lilacinus*, this pathogen is widespread in the environment and can lead to a range of infections, from superficial skin lesions to invasive diseases. This article presents a case of deep cutaneous hyalohyphomycosis caused by *P. lilacinum* in a liver transplant patient, followed by a review of the literature focusing on new antifungal agents. Methods: We reported a brief case description followed by a narrative review of the literature regarding *P. lilacinum* cutaneous and lymphangitic infections in immunocompromised patients. Results: We conducted a review of the literature over the past 20 years, focusing on the clinical features, diagnostic challenges, and therapeutic outcomes of cutaneous and lymphangitic *P. lilacinum* infections in immunocompromised hosts. Conclusions: This review highlights the critical importance of early diagnosis through the analysis of biopsy samples using standard microbiological and histological techniques, complemented by innovative molecular biology methods. We also emphasise the role of appropriate antifungal treatment, despite the absence of an established standard of care, particularly in high-risk patients. Furthermore, we review and discuss the current lack of a standardised therapeutic regimen and the potential of novel antifungal agents as promising treatment options for *P. lilacinum* infections.

## 1. Introduction

*Purpureocillium lilacinum* (*P. lilacinum*), previously known as *Paecilomyces lilacinus* [1], is a filamentous fungus emerging as a human pathogen belonging to hyalohyphomycetes. It is characterised by hyaline septate hyphae (i.e., without pigment in the wall) observed in the tissue of the organs involved, especially skin, eye, lung, or multisite in systemic infections. *P. lilacinum* is ubiquitous and can be found in a wide range of habitats (for example, soil, forests, grassland, and deserts). It has been employed in farming as a biological control of parasitic infestation of plants [2]. *P. lilacinum* has also been described as a hospital indoor contaminant, but the real impact as a source of fungal infections is still not completely clear [3]. Many factors seem to play a role in the emerging importance of saprophytic fungi as human pathogens, including climate change and an increasingly immunosuppressed population. Many authors have hypothesised that global warming is expanding the geographic epidemiology of fungal disease and selecting species with adaptive thermotolerance for mammals [2]. An increasing number of human hyalohyphomycosis caused by this fungus has been described in the last twelve years. *P. lilacinum* is usually an opportunistic pathogen that affects the immunocompromised population. Patients suffering from haematological and oncological conditions seem to have the highest risk of infection, with acute leukaemia being a major risk factor. Moreover, solid organ transplant, diabetes mellitus, and chronic steroid treatment have also been described as common conditions in patients affected by *P. liliacinum* infections [4]. However, a rising number of case reports have described *P. lilacinum* infections among immunocompetent patients without clear risk factors for invasive fungal infection [5]. Interestingly, some activities or jobs, such as farming or gardening, might represent a particular risk for *P. lilacinum* infections [4]. Fungal infections may present from a superficial localised skin phenomenon to an invasive fungal infection. *Purpureocillium lilacinum* infections usually involve skin and soft tissues, but pulmonary involvement has also been described in many case reports [6]. Less common bloodstream infections (including rare cases of endocarditis), sinus infections, and disseminated infections have been reported [7]. Ocular involvement has also been described in the literature [8]. Immunosuppressed patients have a greater risk of invasive or disseminated manifestations. Cutaneous infections due to *P. lilacinum* represent a rare but potentially life-threatening condition in immunocompromised patients. To date, no standard treatment regimen has been established for *Purpureocillium lilacinum* infections. Voriconazole and posaconazole are reported as in vitro active agents and considered reasonable first-line options [9]. Terbinafine has occasionally been reported in the literature as a supportive agent in combination with azoles [4]. In contrast, echinocandins have shown variable susceptibility, while most strains demonstrate resistance to itraconazole and amphotericin B [10]. Herein we report a case of deep cutaneous hyalohyphomycosis caused by *P. liliacinum* with lymphangitic dissemination in a liver transplant patient. Moreover, we reviewed the cases previously reported in the literature among immunocompromised hosts in the last 20 years, aiming to describe clinical presentation, diagnosis, and treatment across the literature.

## 2. Materials and Methods

**Figure 1:**
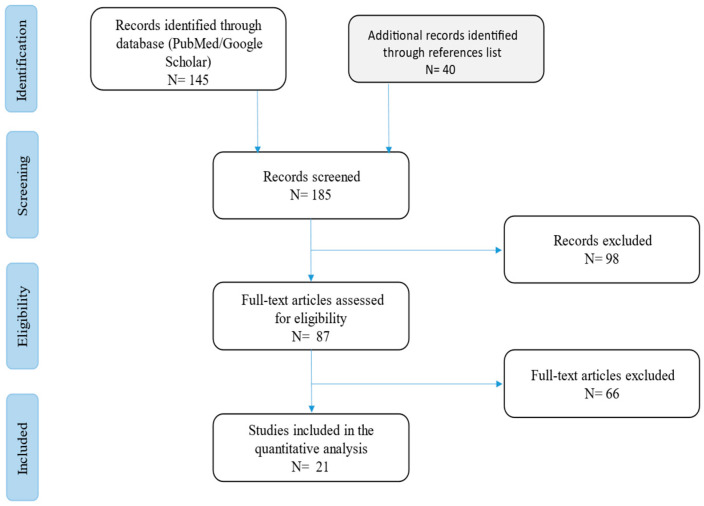
Flow chart of the studies revised in the narrative review.

The current narrative review followed the Scale for the Assessment of Narrative Review Articles (SANRA) flow-chart [11] (Figure 1).

The main aim of this work was to summarise current evidence on cutaneous *Purpureocillium lilacinum* infection in immunocompromised patients, focusing on clinical characteristics and outcomes in this population. A search was run on Google Scholar and PubMed using the terms (‘*Purpureocillium lilacinum*’ [Mesh]) AND (‘Skin’ [Mesh]) AND (‘infection’ [Mesh]) in English. Results were limited to those published between 2004 and 2024. Studies were filtered for practice guidelines, guidelines, meta-analyses, systematic reviews, narrative reviews, case series, and case reports. In addition, we filtered for results including only humans, patients >18 years old, and immunosuppressed patients. Our search strategy permitted the identification of 185 papers, of which 98 were excluded following title and abstract evaluation. Then, the reviewers studied titles and abstracts. Subsequently, 21 papers were included. Researchers reviewed the summaries of all articles found and ultimately used data from the full articles to compile this review paper. Researchers assessed the inclusion of all titles and abstracts without language limitations in English. We duplicated other studies previously included and excluded papers with no methods described, along with papers not strictly related to the aim of the study, and filtered according to journal importance and the number of references. We performed descriptive statistics on the entire study population. Data were analysed using standard statistical methods. Variables were described with medians, absolute values, and rates. Moreover, we have described a case report of an immunocompromised patient admitted to our hospital for *Purpureocillium lilacinum* infection.

## 3. Case Report

**Figure 2:**
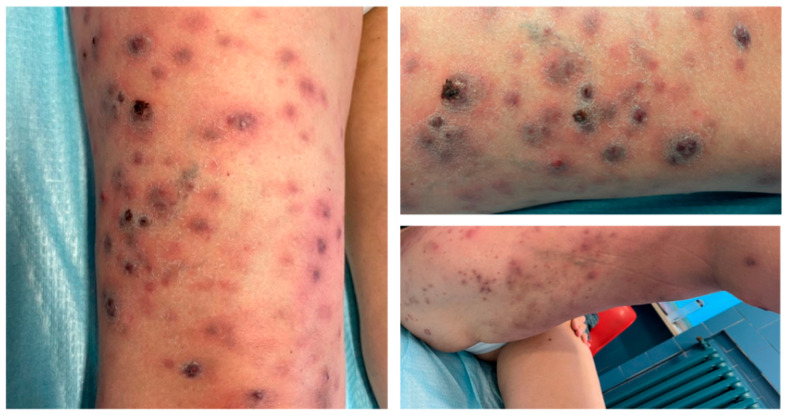
Multiple, monolateral, erythematous-papulo-nodular lesions on the right leg.

A 61-year-old female patient was referred to our department for the presence of erythematous-papulo-nodular lesions on her right leg, as reported in Figure 2.

**Figure 3:**
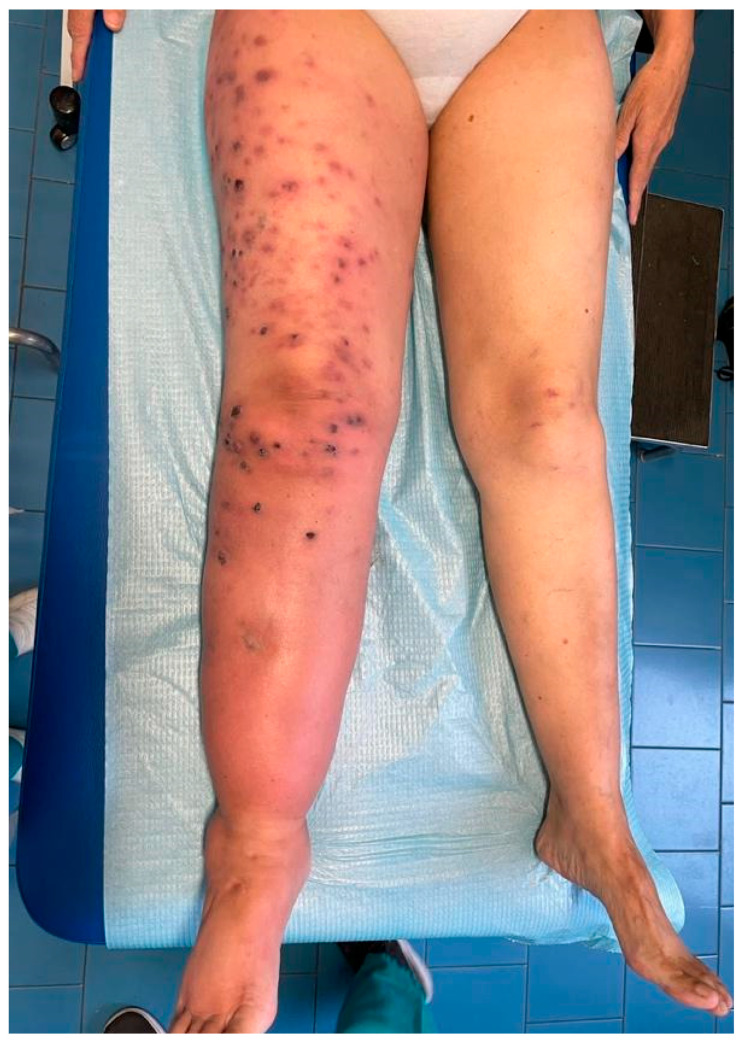
Skin lesions were associated with diffuse soft tissue swelling and redness, referred to as lymphedema.

She had a history of orthotopic liver transplant (OLT) because of autoimmune hepatitis. She underwent a first OLT in 2005 and subsequently a second OLT in 2010 for a graft failure. A third transplant was completed in 2019 for a new graft failure, and the patient was maintained on an immunosuppressive regimen with tacrolimus, mycophenolate mofetil, and oral steroids (i.e., prednisone 10 mg q24h). At the presentation, the patient complained about loss of weight (i.e., about 10 Kg in the last 12 months) and low-grade fever. The lesions were previously diagnosed as cutaneous herpes zoster, and the patient received a seven-day course of oral valacyclovir treatment (i.e., 1 g every 8 h) without improvement. Over the following weeks, lesions gradually spread to form dark violet papulo-nodular elements, some covered by sero-hemorrhagic crusts. Diffuse soft tissue swelling and redness, referred to as lymphedema, were associated with the lesions, as reported in Figure 3. A whole-body CT scan was performed, and no other organ involvement was ruled out.

**Figure 4:**
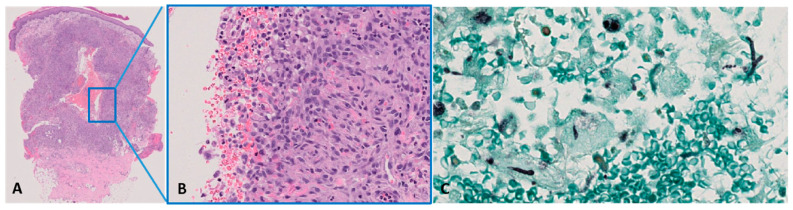
Histopathological features: (**A**,**B**) Granulomatous dermatitis at incisional biopsy (haematoxylin–eosin stain, magnification 10×—(**A**) and 400×—(**B**)). (**C**) Fungal globose yeast-like structures and septate hyphae in the dermis (Grocott stain, magnification ×400).

Patient denied history of a recent trip abroad, animal bites, or tick exposure. Moreover, the patient reported that she lives in the countryside, owns two cats, and loves gardening. She reported a probable skin trauma on her right leg while gardening, some weeks before hospital admission. An empiric antibiotic therapy with doxycycline was started, according to the hypothesis of bacillary angiomatosis, with minimal improvement. *Bartonella henselae* and *Human Herpesvirus 8* serologies and the PCR test were negative. QuantiFERON for *Mycobacterium tuberculosis*, serum galactomannan, and *Cryptococcus* antigen detection were not diagnostic. The blood count analysis found neutrophilic leukocytosis (13 × 10^9^/L) and elevation of the inflammatory index (C-reactive protein 82.8 mg/L, normal range < 5 mg/L). Significant elevation of Beta-D-glucan values was registered in two non-consecutive samples (323 and >600 pg/mL, normal range < 7 pg/mL). Bacterial cultures were negative. Direct microscopic examination from the swab of the ulcerated nodule showed hyaline, septate hyphae and branched conidiophores with stripes. Sabouraud dextrose agar culture was positive for *P. lilacinum*. In parallel, Periodic acid–Schiff (PAS) and Grocott’s staining of skin biopsy identified broad-based hyphae and spores, including rare coccoid elements with a thick PAS-positive capsule, supporting the diagnosis of fungal infection (Figure 4).

**Figure 5:**
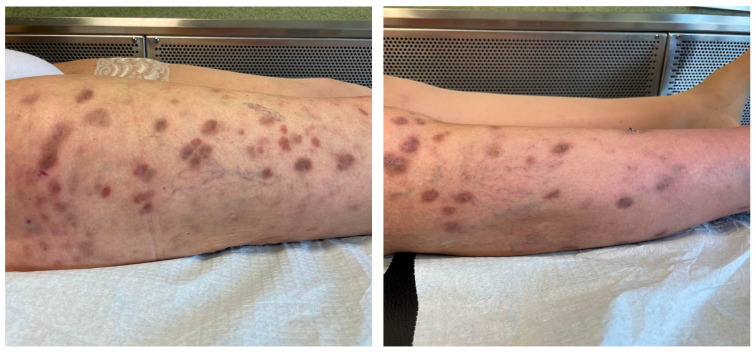
Progressive resolution of skin lesions under treatment after 3 months of therapy.

Antifungal susceptibility tests were not performed. The patient was hospitalised and treatment with terbinafine 250 mg/day plus voriconazole 400 mg/day was administered, with serial azole therapeutic drug monitoring (TDM) due to advanced cirrhosis. CT chest, nasal endoscopy, and blood cultures excluded secondary localisations. Therapy was continued based on clinical response and the reduction in Beta-D-glucan serum levels. Therapy with Voriconazole and terbinafine was withdrawn after nine months, following complete clinical response and healing of the lesions (Figure 5).

Therapy with voriconazole and terbinafine was administered for a total of 9 months, with progressive resolution of the skin lesions.

## 4. Results

The search strategy identified 185 articles, of which 21 studies were included for the final analysis, with a total of 21 patients, and the data are shown in Table 1.

Most of the patients were male (16–76.2%), with a median age of 59 years (range 28–84). Regarding risk factors for fungal infections, frequently reported conditions were steroid use (9–40.9%) and solid organ transplantation (9–40.9%), followed by haematological disorders (3–13.6%). One case involved a patient living with HIV. The majority of patients had no history of exposure or hobbies previously associated with *Purpureocillium lilacinum* infections in the literature.

More frequently, patients presented with nodular lesions (10–45.4%) or papules/pustules (9–40.9%). Three cases presented with erythematous purpuric plaques (13.6%), and one case reported another type of skin manifestation (0.5%).

Painful lesions were reported in around half of the patients (10–45.5%), in nine cases the skin manifestations were painless (40.9%), and in three cases it was not reported.

Regarding therapy, all patients received azoles at a certain point of the treatment (22–100%). Other molecules prescribed were liposomal amphotericin B (4–18.1%) and echinocandins (3–13.6%).

Terbinafine was prescribed in three cases (13.6%), always in combination with azoles.

In two cases, a surgical debridement of the skin lesion was performed (9%).

We found that in 11 cases, patients suffered from local dissemination of infection (50%), and in 5 cases (22.7%), they suffered from systemic involvement. Three patients died before completing treatment (13.6%). Data described in the studies reviewed are summarised in Table 2.

## 5. Discussion

In this study we have reported a case of *P. lilacinum* invasive infection in an OLT recipient, including a revision of the literature of systemic infections due to *P. lilacinum* in the immunocompromised population, focusing on cutaneous fungal involvement and presentations. Moreover, we have summarised new evidence regarding antifungal agents in the pipeline of treatment against *P. lilacinum.* Our case reported the history of a middle-aged female. Median age at diagnosis reported in the literature (ranging from 28 to 84 years old) was in line with that reported in our experience (61 years old), although that literature review showed a prevalence of patients of male sex [12,13,14,15,16,17,18,19,20,21,22,23,24,25,26,27,28,29,30,31,32]. Differences in sex have a determining importance on the prevalence and incidence of many infectious diseases. In an interesting recent review by Egger et al. on invasive fungal infections, notably, aspergillosis, mucormycosis, cryptococcosis, coccidiomycosis, histoplasmosis, and blastomycosis were overrepresented in males, concluding that biological sex should be further investigated as a predisposing factor for fungal infections [33]. In our case, the patient reported a probable skin trauma that had occurred when gardening some weeks before clinical presentation. *P. lilacinum* is a filamentous fungus widely distributed in the environment, including in soil, water, decaying vegetation, insects, and nematodes [1], and can cause human infections by inhalation or direct tissue inoculation [4]. Heutte et al. reported less frequent hospital-acquired infections due to contaminated medical devices, tattoo-related infections, or skin lotion contamination [3], but gardening and farming have been reported [12,13,14,15,16,17,18,19,20,21,22,23,24,25,26,27,28,29,30,31,32] as possible risk factors, such as for the patient in our case report.

Similarly to other fungal infections, immunocompromised patients are at higher risk of *P. lilacinum* infections. Cutaneous and subcutaneous infections represent the most commonly reported clinical manifestations of *P. lilacinum* in the literature [4], as in our case. However, particularly in immunocompromised patients, *P. lilacinum* can occasionally disseminate haematogenously, leading to severe invasive infections such as endocarditis, pneumonia, and central nervous system involvement [7]. We found that infections were mostly localised on the extremities and lesions were usually unilateral, reinforcing the hypothesis that skin inoculation could be an important means of infection, such as in the case we reported [12,13,14,15,16,17,18,19,20,21,22,23,24,25,26,27,28,29,30,31,32]. In the vast majority of cases, the skin findings were characterised by erythematous lesions, nodules, or papules, consistent with what was observed in the case we described [12,13,14,15,16,17,18,19,20,21,22,23,24,25,26,27,28,29,30,31,32]. Among immunocompromised patients, solid organ transplantation and chronic steroids therapy were the most-reported causes of immunosuppression (83%). In this group, deeper and painful skin lesions were observed, such as nodules, or even cellulitis and necrotic ulcerations because of angioinvasion. A bioptic approach was almost always the key to reaching a diagnosis. Definitive diagnosis was mainly established via fungal culture and histology. *P. lilacinum* grows rapidly on Sabouraud agar, ranging in colour from violet to bluish brown. Histological examination usually shows hyphae, phialides, and conidia, which may sporulate in infected tissues [9].

Moreover, molecular identification nowadays plays an important role in species identification. *P. lilacinum*’s reclassification in a genus apart from *Paecilomyces*, on the basis of phylogenic studies [1], may explain significant differences in resistance to most antifungals, including amphotericin B, fluconazole, itraconazole, flucytosine, and the echinocandins [9,10].

There has been no standard of care established up to now, besides that voriconazole or posaconazole should be a reasonable first-line treatment. Terbinafine was reported as a sparring treatment in combination with azoles in the literature reviewed [12,13,14,15,16,17,18,19,20,21,22,23,24,25,26,27,28,29,30,31,32]. Moreover, echinocandins reported a variable susceptibility, and most strains were resistant to itraconazole and amphotericin B [9,10].

Although 37% of patients have been treated with combination therapy in the literature, there is insufficient data to determine whether this is superior to monotherapy. The optimal duration of treatment remains undefined, and surgical debridement should be considered when feasible. Infections in immunocompromised patients are particularly challenging, and current evidence is insufficient to clearly identify the lesion characteristics that indicate resolution of the infectious process. Therefore, based on the available literature, it appears reasonable to continue treatment until all lesions have completely resolved. Resolution or improvement of immunosuppression and surgical source control improve the efficacy of medical treatment [12,13,14,15,16,17,18,19,20,21,22,23,24,25,26,27,28,29,30,31,32]. In our case, 1–3 Beta-D-glucan serology was used to monitor clinical response, but we found no evidence to support this practice in the current literature [12,13,14,15,16,17,18,19,20,21,22,23,24,25,26,27,28,29,30,31,32]. Mortality is around 13%, and with the limitations of a low number of studies, there does not seem to be a correlation with particular predisposing factors.

Recently, new antifungals have been studied. Ibrexafungerp is the first representative of a novel class of structurally distinct glucan synthase inhibitors: triterpenoids. Although it has shown excellent activity in vitro against *Candida* spp., *Aspergillus* spp., and some rare moulds, the activity against *P. lilacinum* is negligible [34]. Fosmagenopix is a new oral antifungal whose mechanism of action is targeting the GWT1 enzyme required for the localization of glycosylphosphatidylinositol-anchored mannoproteins in fungi. It has shown great activity in vitro against *P. lilacinum*, with MICs lower than those of posaconazole and voriconazole [35]. Olorofim selectively inhibits fungal dihydroorotate dehydrogenase (DHODH), a key enzyme in the pyrimidine biosynthesis pathway. For Olorofim, despite few data available, no in vitro efficacy has been observed in *P. lilacinum* clinical isolates, whereas MICs for *P. variotii* were low [34]. With regard to Rezafungine, a next-generation, broad-spectrum, and long-lasting echinocandin, there are no data in the literature concerning efficacy on *P. lilacinum* at this moment.

In conclusion, the review emphasises the critical need for early diagnosis and appropriate antifungal treatment, particularly in high-risk patients, accompanied by surgical debridement when feasible. Data on treatment duration and the superiority of combination therapy with respect to monotherapy are lacking, despite the fact that new, recently approved antifungals present efficacy and safety profiles that are promising for the treatment of this rare infection.

## Figures and Tables

**Table 1:** Literature regarding cutaneous infection due to *P. lilacinum* in immunocompromised hosts.

	Age Sex	Comorbidities	Hobby	Skin Lesions	Pain	Site	Diffusion	Therapy	Outcome	REF
1	79, M	Diabetes mellitus; Pemphigus vulgaris	Gardening	Erythematous pustule, warm, single	no	Hand, monolateral	Bilateral leg and ankle cellulitis, encephalic	Itraconazole 200 mg bid Voriconazole 6 mg/kg every 12 h	Dead	[12]
2	84, M	Steroid therapy; Diabetes mellitus	NA	Swelling and bursitis of the first metatarsophalangeal joint	yes	Lower extremity, monolateral	No	Surgery plus voriconazole 200 mg bid	Recovered	[13]
3	65, M	Steroid therapy for temporal arteritis	NA	Erythematous and oedematous area with papules and pustules	no	Lower extremity, monolateral	Cellulitis	Itraconazole 200 bid plus surgery	Recovered	[14]
4	56, M	Liver transplant	NA	Erythematous, tender single nodule	no	Knee, monolateral	Multiple nodules following lymphatic distribution	Voriconazole 200 mg bid alone and in association with Terbinafine Voriconazole 300 mg bid	Recovered	[15]
5	72, M	Renal transplant	NA	Erythematous nodule	no	Forearms, monolateral	Multiple reddish nodules	Itraconazole 200 mg daily Voriconazole 400 mg/day and Terbinafine 250 mg/day	Recovered	[16]
6	28, M	Bone marrow transplant; Previous *Pseudomonas aeruginosa* necrotising fasciitis on the face	None	Erythematous papules and pustules, crusted at the margin of previous ulcers	no	Face and site of closed tracheostomy	Multiple necrotising ulcers	Itraconazole 200 bid Micafungin 50 mg/day LAMPHB 150 mg/day Voriconazole 400 mg/day	Recovered	[17]
7	74, F	Immunosuppressed therapy for autoimmune haemolytic anaemia	NA	Erythematous brownish plaque, single	yes	Hemiface	Multiple papules and abscesses around the plaque on the hemiface	Griseofulvin 500 mg/daily Itraconazole 200 mg/day for 11 weeks, then 400 mg daily, 7 days a month	Recovered	[18]
8	71, F	Steroid therapy for giant cell arteritis	NA	Erythematous nodule	no	Elbow, monolateral	NA	Micafungin 50 mg/day Itraconazole *(dosage not available)*	NA	[19]
9	52, M	Renal transplant	Gardening	Oedematous and erythematous area	no	Lower extremity, monolateral	Cellulitis with multiple fistula orifices	Posaconazole 300 mg/day Surgery for local relapse	Recovered	[20]
10	59, M	Renal transplant	NA	Erythematous papules, pustules and small ulcers	yes	Foot and interdigital space, monolateral	Multiple lesions on foot and leg, sepsis due to *Candida* spp. and Gram-negative bacteria	Itraconazole 400–600 mg/day Voriconazole 400 mg/day LAMPHB	Dead	[21]
11	50, M	CLL; previous pyoderma gangrenosum	NA	Purpuric nodules and a necrotic ulcer	yes	Lower extremity, monolateral	Extended necrotic ulcer	LAMPHB 6 mg/kg/day Micafungin 150 mg/day Voriconazole 400 mg bid Isavuconazole IV 372 mg/day plus surgery	Recovered	[22]
12	33, M	Renal transplant	Recent tattoo	Erythematous papules, pustules clustered over tattoo	no	Left forearm and shin, right ankle	None	Voriconazole 400 mg/day	Recovered	[23]
13	63, M	Heart transplantation; Diabetes mellitus	NA	Erythematous nodules	yes	Leg and elbow	Multiple necrotic ulcers	Voriconazole 400 mg/day Intralesional Voriconazole (1 mL) followed by oral treatment with Terbinafine 250 mg a day the first month, then 125 mg/day	Recovered	[24]
14	60, M	Pulse steroid therapy for rheumatoid arthritis	NA	Erythematous nodules with pseudo-verrucous aspect	NA	Hemiface	Multiple necrotic ulcers on face and hand	Voriconazole 400 mg/day Posaconazole 400 mg/bid	NA	[25]
15	48, F	Renal transplant	NA	Erythematous oedematous area	yes	Lower extremity, monolateral	Extensive cellulitis with multiple ulcers	Itraconazole 400 mg/day Voriconazole (dosage non reported)	Recovered	[26]
16	40, M	AIDS	NA	Erythematous nodules	yes	Lower extremity, monolateral	Multiple ulcerated nodules on entire leg and body	Itraconazole 200 mg bid LAMPHB 5 mg/kg/day Voriconazole 200–300 mg/bid	Dead	[27]
17	69, M	Liver transplant recipient	None, lives with a dog	Erythematous nodules with pseudo-verrucous aspect	yes	Hand, monolateral	Multiple nodules tracking on arm and axilla	Voriconazole 400 mg/day	Recovered	[28]
18	53, M	Steroid therapy for autoimmune haemolytic anaemia	None	Erythematous pustule and ulcerated nodule	no	Neck	None	Itraconazole 400 mg/day Posaconazole 300 mg/die	Recovered	[29]
19	67, F	X-linked chronic granulomatous disease	Sheep farmer	Erythematous nodules with pustules	yes	Upper extremity (thumb)	None	Voriconazole 400 mg/day	Recovered	[30]
20	51, F	Steroid therapy for nephrotic syndrome	NA	Recurrent papules, pustules, and ulceration	NA	Finger and forearm, monolateral	None	Voriconazole (dosage not available)	Recovered	[31]
21	NA, M	Steroid therapy for Evans’ Syndrome	Farmer	Erythematous papules and pustules	yes	Forearm, monolateral	Multiple ulcerated lesions	Voriconazole 400 mg/day then Posaconazole 300 mg/day with surgery	Recovered	[32]

NA: not reported; LAMPHB: liposomial amphotericin B.

**Table 2:** Characteristics of immunocompromised patients with cutaneous infections caused by *P. lilacinum*.

	n (%)
Patient age	
Years, mean (min-max)	59 (28–84) years
Sex	
Male	16 (76.2)
Female	6 (27.2)
Race/ethnicity	
Caucasian	9 (40.9)
Black/African American	6 (27.2)
Asiatic	7 (31.8)
Immunosuppression	
Steroids	9/22 (40.9)
Solid transplant	9/22 (40.9)
Haematological condition	3/22 (13.6)
HIV/AIDS	1/22 (0.5)
Hobbies	
Unknown/Other	15/22 (68)
Farmer	3/22 (13.6)
Gardening	2/22 (9)
Skin lesions	
Papules/pustules	9/22 (40.9)
Nodules	10/22 (45,4)
Erythematous purpuric plaques	3/22, 13.6)
Other	1/22 (0.5)
Localization	
Upper extremities	7/22 (31.8)
Lower extremities	10/22 (45.4)
Pain associated	
Yes	10/22 (45,4)
No	9/22 (40.9)
Local diffusion	11/22 (50)
Systemic diffusion/secondary foci	5/22 (22.7)
Treatment	
Azoles	22/22 (100)
Terbinafine	3/22 (13.6)
Echinocandins	3/22 (13.6)
Liposomal amphotericin	4/22 (18.1)
Griseofulvin	1/22 (0.5)
Surgical debridement	2/22 (9)
Mortality	3/22 (13.6)

## Data Availability

Data are available upon reasonable request.
